# Simple and fast prediction of Legionella sp. in community-acquired pneumonia: validation of a prediction rule

**DOI:** 10.1186/cc11977

**Published:** 2013-03-19

**Authors:** S Haubitz, F Hitz, L Graedel, T Wiemken, P Peyrani Dicastelnuovo, J Ramirez, M Batschwaroff, C Fux, B Mueller, P Schütz

**Affiliations:** 1 Kantonsspital Aarau, Switzerland; 2 University of Basel, Switzerland; 3 University of Louisville School of Medicine, Louisville, KY, USA

## Introduction

Ruling out Legionella sp. in patients presenting with community-acquired pneumonia (CAP) is important due to differences in treatment regimens. Yet antigen tests as well as blood cultures have low sensitivity and an important time delay, making empirical broad spectrum coverage necessary particularly in severe cases. Fiumefreddo and colleagues recently proposed a clinical score based on six clinical and laboratory variables (fever, cough, sodium, lactate-dehydrogenase, C-reactive protein, platelet count) which allowed assessing the likelihood of Legionella [1]. Yet these variables need validation in an independent patient cohort before implementation into clinical routine.

## Methods

We analyzed data from a large multinational database of patients with CAP (CAPO) [2] between 2001 and 2012. We performed logistic regression analysis and the area under the receiver operating characteristics (AUC) curve to study the association of these variables with the diagnosis of Legionella.

## Results

Data for 8,278 CAP patients were analysed; the infectious organism was known in 2,321 cases (28%), including a total of 101 patients with urinary antigen-confirmed Legionnaires' disease and 983 patients with confirmed pneumococcal disease. All variables were predictors for Legionella with odds ratios ranging from 1.002 to 5.767. Combining the variables in a joint logistic regression model showed a high predictive accuracy with an AUC of 0.86.

## Conclusion

This analysis validates the Legionella score in an independent sample and shows high diagnostic accuracy. Interventional trials with adapted antibiotic regimes for non-inferiority in a real live population are warranted.
